# An Epitome of the History of Medicine

**Published:** 1899-03

**Authors:** 


### Reviews of Books.

**An Epitome of the History of Medicine.** By ROSWELL PARK, M.D. Pp. xiv., 348. Philadelphia: The F. A. Davis Company. 1897.

It is with very great pleasure that we have read Dr. Roswell Park's praiseworthy book. We congratulate the University of Buffalo on having authorised the delivery of the course of lectures upon which this work was founded, and upon having a Professor who, in addition to the work of his own special department, was capable of undertaking so serious an additional responsibility. A succinct, but fairly complete, history of medicine from the earliest times to the present day has been compiled by the author, special chapters being devoted to the history of the development of antiseptic surgery, American medicine, anæsthesia, and dentistry. The book gives proof of much investigation and study on the author's part, and is written in good nervous English: the author however is obviously more conversant with the history of modern than of ancient medicine, and the hypercritical critic may succeed in discovering an occasional slip; *e.g.* (p. 34) "Aretæus, who died about 170 B.C., was one of the most brilliant lights of antiquity previous to the Christian era. . . . He came from Cappadocia about the end of the reign of Nero, and lived in Alexandria." It is still a reproach to the Anglo-Saxon nations that so little attention has been paid among them to the history of the medical art, and a really adequate work on the subject does not exist in their language. The author has with much industry done something to remove that reproach, and for his accomplishment we congratulate and thank him.

